# Distinct Modulatory Effects of Fever-Range Hyperthermia on the Response of Breast Cancer Cells and Macrophages to Mistletoe (*Viscum album* L.) Extract

**DOI:** 10.3390/ph14060551

**Published:** 2021-06-08

**Authors:** Henryk M. Kozłowski, Małgorzata Pawlikowska, Justyna Sobocińska, Tomasz Jędrzejewski, Artur Dzialuk, Sylwia Wrotek

**Affiliations:** 1 Department of Immunology, Faculty of Biological and Veterinary Sciences, Nicolaus Copernicus University, 1 Lwowska Str., 87-100 Torun, Poland; m_pawlikowska@umk.pl (M.P.); j.sobocinska@umk.pl (J.S.); tomaszj@umk.pl (T.J.); wrotek@umk.pl (S.W.); 2 Department of Genetics, Faculty of Biological Sciences, Kazimierz Wielki University, 10 Powstańców Wielkopolskich Ave., 85-090 Bydgoszcz, Poland

**Keywords:** mistletoe extract, hyperthermia, cytokines, fever, inflammation, reactive oxygen species, cell cycle distribution

## Abstract

Heat utility as a critical component of fever is often ignored, although the symptom is observed in many medical conditions. Mistletoe extract (ME) is an adjunctive medication prescribed to cancer patients. The increase in body temperature is frequently observed in patients following ME administration. Nevertheless, the impact of this fever on the effectiveness of therapy is unknown. Therefore, we aimed to investigate the effect of fever-range temperatures on ME-treated breast cancer cells and macrophages. The cells were simultaneously stimulated with ME and subjected to fever-range hyperthermia (FRH; 39 °C or 41 °C). After co-treatment, the cell viability, generation of reactive oxygen species (ROS), cell cycle distribution, and production of pro-inflammatory factors (interleukin (IL)-1β, IL-6, and cyclooxygenase (COX)-2) were evaluated. The results showed that the exposure of ME-treated breast cancer cells to FRH at 39 °C resulted in a slight decrease in their viability, whereas FRH of 41 °C enhanced this effect. Only FRH of 41 °C induced minor changes in ROS level in ME-treated breast cancer cell lines. In ME-treated macrophages, FRH stimulated cell proliferation. The cell cycle distribution analysis showed a difference between cells cultured at 39 °C and 41 °C in all examined cell lines. Moreover, hyperthermia at 41 °C completely inhibited the ME-induced increase in IL-1β and IL-6 expression in MCF-7 breast cancer cells, whereas this effect was not observed in 4T1 breast cancer cells. In contrast, in ME-treated macrophages, FRH of 41 °C strongly up-regulated expression of the pro-inflammatory factors. We conclude that fever is an important component of ME therapy that differentially affects cancer and immune cells.

## 1. Introduction

Breast cancer is the most common cancer and also the primary cause of mortality due to cancer in females around the world. In spite of many improvements in the use of hormonal and adjuvant cytotoxic therapies for breast cancer patients, the reduction in mortality is still not satisfactory [1].

Mistletoe extract (ME) is the most frequently prescribed therapy as an adjunct to standard treatment regimens for various malignancies in Europe [2,3]. It is widely used based on its ability to reduce emotional stress. However, it is also an immunostimulant, and its anticancer properties have been confirmed in experimental studies as well as clinical trials [3,4].

ME is generally well tolerated, although some common side effects such as local reactions at the injection site (e.g., redness, swelling, itchiness) and mild flu-like symptoms including fever have been described [5,6,7,8,9,10]. Although fever was observed in a large group of ME-treated patients [10], little is known about its significance in ME therapy [11], and the direct functional consequences of ME-induced body temperature increase are unclear. Thus, it is not known whether ME-induced fever is only a side effect, which can be inhibited, or is a pivotal factor that acts synergistically with ME.

According to published data, the increase in body temperature is induced by the binding of some ME compounds, such as lectins, to the pattern recognition receptors on immune cells [11,12]. Then, the signal is transmitted, and the expression of pyrogenic cytokines such as interleukin (IL)-1β, IL-6, and tumor necrosis factor α (TNF-α) is observed. These cytokines activate cyclooxygenease-2 (COX-2), which is an enzyme involved in prostaglandin E2 production (PGE2), which is a final mediator of fever [13,14]. Our previous research on mice with severe combined immunodeficiency (SCID) showed that in the process of fever induction, the involvement of the innate immune cells such as macrophages, rather than lymphocytes, is needed [15]. Since macrophages secrete many factors including cytokines and reactive oxygen species (ROS) that create a microenvironment for tumor cells, we wanted to investigate whether various thermal conditions may affect their response to ME. Additionally, due to the unresolved question as to whether fever may support anticancer ME therapy, we decided to explore the effects of heat on cancer cells directly. The aim of our study was to examine whether fever-range temperatures (above 37 °C) modify the effects triggered by ME therapy in macrophages and breast cancer cells. To resolve this problem, we utilized febrile-range hyperthermia (FRH), which is considered to be a surrogate for fever in in vitro experiments.

We found that FRH modulates cell viability, ROS generation, cell cycle arrest, and the expression of inflammatory markers (i.e., pro-inflammatory cytokines and COX-2) in breast cancer cells and in macrophages. The effect was dependent on the level of FRH, and FRH exerted different effects on ME-treated breast cancer cells and macrophages.

## 2. Results

### 2.1. Exposure to Heat at 41 °C Decreased Viability of ME-Treated Mcf-7 Cells

**Figure 1:**
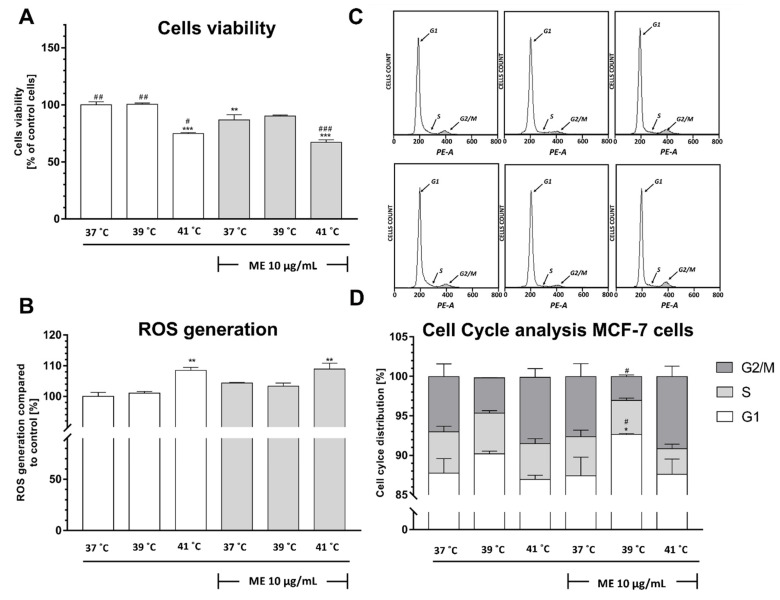
Effect of heat exposure on the viability, as measured using the MTT test (**A**), ROS production, as measured using carboxy–H_2_DCFDA staining followed by flow cytometry analysis (**B**), and cell cycle distribution, as measured using propidium iodide staining followed by flow cytometry analysis (**C**,**D**), of ME-treated MCF-7 cells. Data are presented as the mean ± S.E.M. of three independent experiments. Asterisks indicate statistical significance in comparison to untreated control cells, and slashes indicate significance in comparison to ME-treated cells cultured at 37 °C (*** or ### *p* < 0.001, ** or ## *p* < 0.01, * or # *p* < 0.05). ME indicates mistletoe extract.

To determine whether thermal conditions affect cell viability in ME-treated breast cancer cells, the MTT assay was conducted. The observation of control MCF-7 cells that were not treated with ME revealed that exposure to heat alone at 41 °C significantly inhibits their proliferation (*p* < 0.001) (Figure 1A). We found that the exposure of MCF-7 cells cultured at 37 °C to ME alone decreased their viability compared to not-treated cells (*p* < 0.01), whereas the exposure of ME-treated MCF-7 cells to heat at 41 °C enhances this reduction in cell viability (*p* < 0.001).

### 2.2. Exposure to Heat at 41 °C Increased Reactive Oxygen Species Production in ME-Treated MCF-7 Cells

Having observed that both not-treated and treated with ME MCF-7 cells cultured at 41 °C display a decrease in cell viability (Figure 1A), we wanted to determine whether this effect is associated with an increase in ROS production. Indeed, we observed a significant increase in ROS generation in cells cultured at 41 °C, regardless of exposure to ME (*p* < 0.01) (Figure 1B). We did not notice any significant changes in the ROS level in ME-treated MCF-7 cells cultured at 37 °C and 39 °C in comparison to control cells (Figure 1B).

### 2.3. ME Combined with Heat Exposure to 39 °C Induced a Significant Cell Cycle Arrest at the G1 Phase in MCF-7 Cells

Since exposure of breast cancer cells to heat at 41 °C is able to decrease their viability (Figure 1A), we decided to check whether these culture conditions modulate cell cycle distribution in the MCF-7 cell line. In ME-treated cells, we did not notice any significant changes in the cell cycle of breast cancer cells cultured at 41 °C as well as cells cultured at 39 °C, in comparison to control, which was not-treated with ME cells (Figure 1C,D). Similarly, in ME-treated MCF-7 cells cultured at 37 °C, we did not observe any significant changes in cell cycle distribution in comparison to control cells. However, the elevation of the culture temperature to 39 °C after ME treatment induced a cell cycle arrest at the G1 phase in comparison to control cells and ME-treated cells cultured at 37 °C (*p* < 0.05 and *p* < 0.05, respectively). Furthermore, in ME-treated cells cultured at 39 °C, we observed a decrease in the percentage of cells in the G2/M phase in comparison to ME-treated cells cultured at 37 °C (*p* < 0.05). Although ME-treated cells exposed to heat at 41 °C displayed a significant reduction in cell viability (Figure 1A), we did not observe any significant changes in cell cycle distribution.

### 2.4. Exposure to Fever-Range Hyperthermia Decreased the Viability of ME-Treated 4T1 Cells

**Figure 2:**
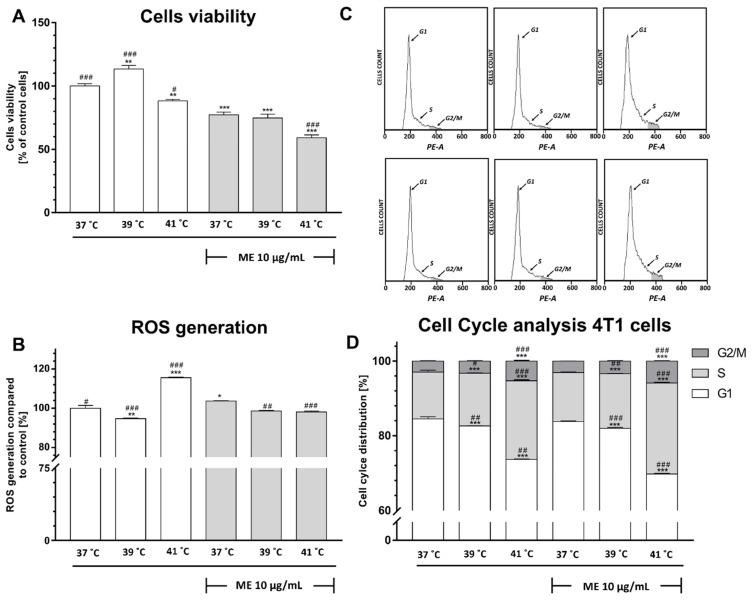
Effect of heat exposure on the viability, measured using MTT test (**A**), ROS production, measured using carboxy-H_2_DCFDA staining followed by flow cytometry analysis (**B**), and cell cycle distribution, measured using propidium iodide staining followed by flow cytometry analysis (**C**,**D**) of ME-treated 4T1 cells. Data are presented as the mean ± S.E.M. of three independent experiments. Asterisks indicate statistical significance in comparison to untreated control cells, and slashes indicate significance in comparison to ME-treated cells cultured at 37 °C (*** or ### *p* < 0.001, ** or ## *p* < 0.01, * or # *p* < 0.05). ME indicates mistletoe extract.

To determine whether thermal conditions affect cell viability in ME-treated 4T1 breast cancer cells, we used the MTT assay. In our control groups, which were not treated with ME, we have observed that exposure to heat alone at 39 °C increases cell viability (*p* < 0.01) (Figure 2A). Nevertheless, the elevation of culture temperature to 41 °C revealed a significant decrease in cell viability (*p* > 0.001). In ME-treated 4T1 cells cultured at 37 °C as well as cultured at 39 °C, we have found a significant decrease in cell viability in comparison to not-treated cells (*p* < 0.001 and *p* < 0.001, respectively). Elevation of ambient temperature to a range of 41 °C potentiates in ME-treated 4T1 cells the effect of reduced cell viability compared to not-treated and ME-treated cells cultured at 37 °C (*p* < 0.001 and *p* < 0.001, respectively).

### 2.5. ME Enhances a Slight Increase in Reactive Oxygen Species Level

Since the exposure of 4T1 breast cancer cells to ME triggered a decrease in cell viability, we wonder whether this effect is associated with ROS production. The observation of our control, not-treated with ME cells, revealed that heat at 39 °C induces a slight, but statistically significant, decrease in reactive oxygen species level (*p* < 0.01) (Figure 2B). In contrast, heat at 41 °C gives rise to ROS levels compared to not-treated cells cultured at 37 °C (*p* < 0.001). ME induces a slight increase in ROS level in breast cancer cells cultured at 37 °C compared to control cells (*p* < 0.05); this effect can be abolished by introducing heat at both examined temperatures 39 °C and 41 °C in comparison to ME-treated cells (*p* < 0.01 and *p* < 0.001, respectively).

### 2.6. ME Combined with Heat Exposure to 41 °C Induced a Significant Cell Cycle Arrest at the S Phase in 4T1 Cells

Having observed that 4T1 cells treated with ME exhibited a significant reduction in cell viability at 37 °C (Figure 2A), we decided to assess whether this effect is associated with a change of cell cycle distribution (Figure 2C,D). Our control groups not-treated with ME have shown that the elevation of ambient temperature to 39 °C caused a slight decrease in cell cycle arrest in the G1 phase associated with a slight increase in cell count in the S phase in comparison to control cells cultured at 37 °C (*p* < 0.001 and *p* < 0.001, respectively). Similarly, the elevation of ambient temperature to 41 °C reduced cells counts in the G1 phase compared to control cells (*p* < 0.001). At the same time, we have noticed an increased number of cells in the S phase as well as in the G2/M phase in comparison to control cells not-treated with ME and cultured at 37 °C (*p* < 0.001 and *p* < 0.001, respectively). Interestingly, we observed that ME reduces 4T1 cell viability at 37 °C, but we did not notice any significant changes in cell cycle distribution after ME treatment. Nevertheless, after the elevation of ambient temperature to 39 °C in ME-treated cells, there was a significant increase in the number of cells in the S phase and decreased count of cells in the G1 phase in comparison to ME-treated cells cultured at 37 °C (*p* < <0.01 and *p* < 0.001, respectively) as well as in comparison to not-treated control cells cultured at 37 °C (*p* < 0.001 and *p* < 0.001, respectively). Furthermore, cells treated with ME and cultured at 41 °C have shown an increased number of cells in S phase and G2/M phase in comparison to ME-treated cells cultured at 37 °C (*p* < 0.001 and *p* < 0.001, respectively) as well as in comparison to not-treated control cells cultured at 37 °C (*p* < 0.001 and *p* < 0.001, respectively). This finding was associated with a significant decrease of cells in G1 phase compared to ME-treated and not-treated cells cultured at 37 °C (*p* < 0.001 and *p* < 0.001, respectively).

### 2.7. Exposure to Temperatures of 39 °C or 41 °C Prevented the ME-Induced Decrease in RAW 264.7 Cell Viability

**Figure 3:**
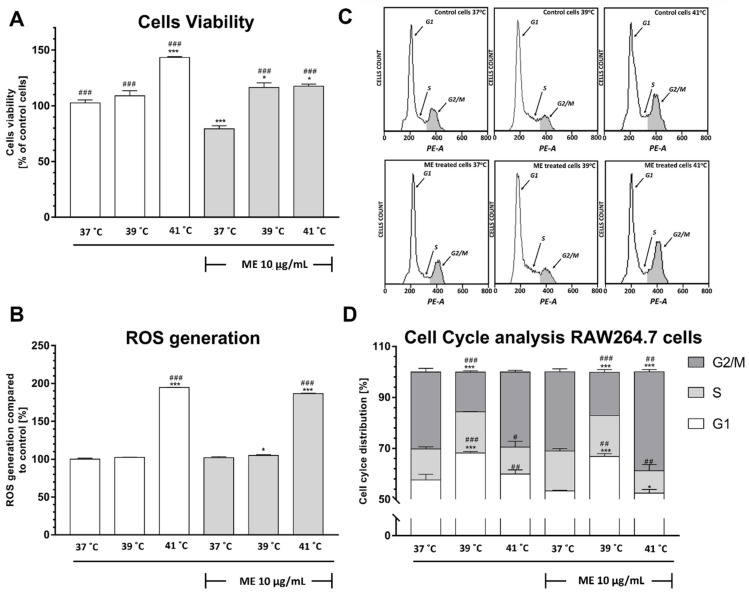
Effect of heat exposure on the viability, measured using MTT test (**A**), ROS production, measured using carboxy-H_2_DCFDA staining followed by flow cytometry analysis (**B**), and cell cycle distribution, measured using propidium iodide staining followed by flow cytometry analysis (**C**,**D**) of ME-treated RAW 264.7 cells. Data are presented as the mean ± S.E.M. of three independent experiments. Asterisks indicate statistical significance in comparison to untreated control cells and slashes indicate significance in comparison to ME-treated cells cultured at 37 °C (*** or ### *p* < 0.001, ## *p* < 0.01, * or # *p* < 0.05). ME indicates mistletoe extract.

To determine whether thermal conditions affect the cell viability in ME-treated RAW 264.7 cells, we conducted an MTT assay. The control RAW 264.7 cells, which were not-treated with ME, revealed that exposure to heat at 41 °C stimulates the proliferation of macrophages (*p* < 0.001), whereas the exposure to heat at 39 °C did not induce any changes in the cell viability in comparison to control cells cultured at 37° C (Figure 3A). We found that the exposure of RAW 264.7 cells cultured at 37 °C to ME significantly inhibited their viability in comparison to untreated cells (*p* < 0.001). This effect can be abolished by elevation of the temperature to 39 °C or 41 °C (*p* < 0.001 and *p* < 0.001, respectively). The elevation of temperature to 39 °C and 41 °C not only abolished this effect, but it also stimulated cell proliferation (*p* < 0.05 and *p* < 0.01, respectively).

### 2.8. Exposure to Heat at 41 °C Increased Reactive Oxygen Species Generation in RAW 264.7 Cells

Since the production of ROS by macrophages as a mechanism to kill tumor cells is well established [16], in this experiment, we wanted to assess whether various thermal conditions affect ROS generation in ME-treated RAW 264.7 cells. The control, not-treated with ME cells, displayed a significant increase in ROS level (*p* < 0.001) after exposure to heat at 41 °C (Figure 3B). The treatment of RAW 264.7 cells with ME did not change ROS levels in cells cultured at 37 °C or at 39 °C. Interestingly, ME-treated cells exposed to 41 °C display two times higher ROS generation than control cells (*p* < 0.001).

### 2.9. ME Treated RAW 264.7 Cells Demonstrate a Significant Arrest in the G2/M Phase after Heat Exposure

Having observed that treatment of RAW 264.7 cells with ME induced a significant reduction in cell viability at 37 °C (Figure 3A), we decided to assess whether this effect is associated with a change of cell cycle distribution (Figure 3C,D). We did not observe any changes in cell cycle distribution in RAW264.7 cells not-treated with ME cultured at 41 °C. Interestingly, we noticed that the elevation of ambient temperature to 39 °C caused a significant increase in cell cycle arrest in the G1 phase associated with a decrease in cell count in the G2/M phase in comparison to control cells (*p* < 0.001 and *p* < 0.001, respectively). Similarly, the elevation of ambient temperature to 39 °C induced in ME-treated cells a cell cycle arrest in the G1 phase and resulted in a significant reduction of cells in the G2/M phase compared to ME-treated cells cultured at 37 °C (*p* < 0.001 and *p* < 0.001, respectively). In our study, we did not find any significant change in cell cycle distribution after ME-treatment at 37 °C. However, cells treated with ME and cultured at 41 °C demonstrated an increase in the number of G2/M cells and a decreased number of cells in the S phase in comparison to not-treated with ME control cells (*p* < 0.001 and *p* < 0.05, respectively). Furthermore, there was a significant increase in the number of cells in the G2/M phase and decreased count of cells in the S phase in comparison to ME treated cells cultured in 37 °C (*p* < 0.01 and *p* < 0.01, respectively).

### 2.10. Exposure to Temperatures of 39 °C or 41 °C Inhibited ME-Induced mRNA Expression of IL-1β and IL-6 in MCF-7 Cells

**Figure 4:**
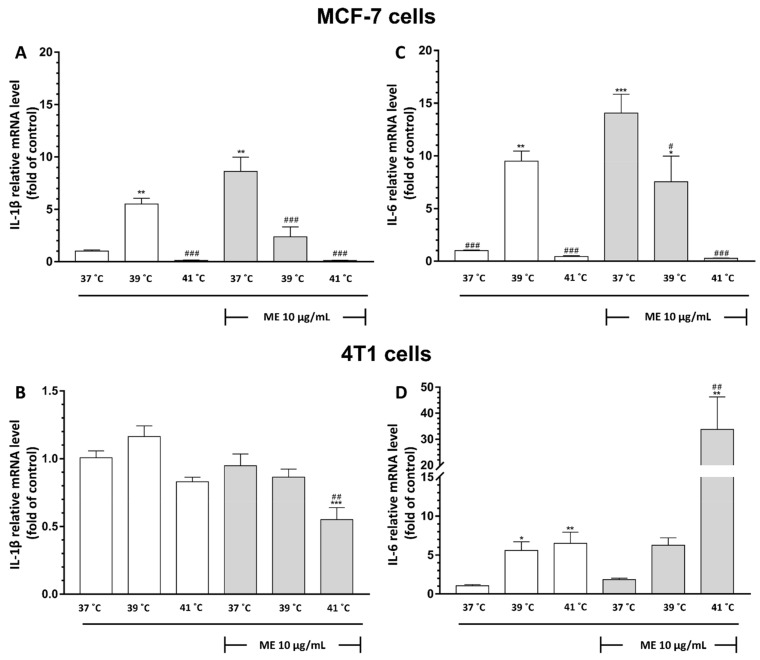
Effect of heat exposure on ME-induced mRNA expression of (**A**,**B**) IL-1β, (**C**,**D**) IL-6 in MCF-7 cells (**A**,**C**), and 4T1 cells (**B**,**D**), respectively. mRNA expression was determined by quantitative real time-PCR. Data are presented as the mean ± S.E.M. of three independent experiments. Asterisks indicate statistical significance in comparison to untreated control cells, and slashes indicate significance in comparison to ME+/37 °C (*** or ### *p* < 0.001, ** or ## *p* < 0.01, * or # *p* < 0.05). ME indicates mistletoe extract.

Since we observed that thermal conditions significantly affect the cell viability, ROS generation, and cell cycle distribution of ME-treated MCF-7 cells, we next wanted to determine whether FRH affects the expression of pro-inflammatory cytokines. The control cells that were not treated with ME but were exposed to heat at 39 °C displayed an increase in mRNA expression of IL-1β in comparison to control cells (*p* < 0.01), whereas exposure to 41 °C did not trigger such an effect (Figure 4A). ME significantly enhanced mRNA expression of IL-1β in MCF-7 cells cultured at 37 °C (*p* < 0.01). Exposure of ME-treated MCF-7 cells to heat at either 39 °C or 41 °C significantly diminished this expression in comparison to the ME-treated cells cultured at 37 °C (*p* < 0.001 and *p* < 0.001, respectively).

The observation of not-treated MCF-7 cells revealed the statistically significant increase in mRNA expression of IL-6 observed only in cells exposed to heat at 39 °C (*p* < 0.01) (Figure 4B). ME treatment enhanced mRNA expression of IL-6 in MCF-7 cells cultured at 37 °C in comparison to control cells (*p* < 0.001). It is noteworthy that we observed a temperature-dependent decrease in mRNA expression in ME-treated cells exposed to heat. The relative expression of IL-6 mRNA in ME-treated cells cultured at 39 °C was almost two times lower than cells treated with ME and cultured at 37 °C. (*p* < 0.05). In ME-treated MCF-7 cells, an exposure to 41 °C caused a complete inhibition of IL-6 mRNA expression compared to ME-treated cells cultured at 37 °C (*p* < 0.001).

### 2.11. Exposure of ME-Treated 4T1 Breast Cancer Cells to Heat at 41 °C Differentially Modulates mRNA Expression of IL-1β and IL-6

Since we observed that heat exposure could modify ME-induced cytotoxicity, ROS generation, and cell cycle distribution, we wonder about the influence of the combination of ME and heat on pro-inflammatory cytokines mRNA expression. Our results have shown that the elevation of ambient temperature to 39 °C and 41 °C alone is not able to change IL-1β mRNA expression in 4T1 cells in comparison to not-treated cells cultured at 37 °C (Figure 4B). Interestingly, ME did not influence IL-1β mRNA expression at 37 °C, but the elevation of ambient temperature to 41 °C of ME-treated cells reduced IL-1β mRNA expression in comparison to ME-treated and not-treated cells cultured at 37 °C (*p* < 0.01 and *p* < 0.001, respectively).

The observation of not-treated with ME 4T1 breast cancer cells revealed a statistically significant increase in IL-6 mRNA expression after the exposure to heat at 39 °C and 41 °C in comparison to cells cultured at 37 °C (*p* < 0.05 and *p* < 0.01, respectively) (Figure 4D). Furthermore, ME-treated cells cultured at 37 °C did not show any significant changes in IL-6 mRNA expression compared to not-treated control cells. Nevertheless, the elevation of ambient temperature to 41 °C of ME-treated cells has shown a significant increase in IL-6 mRNA expression compared to ME-treated and not-treated cells cultured at 37 °C (*p* < 0.01 and *p* < 0.01, respectively).

### 2.12. Exposure to Heat at 41 °C Enhanced mRNA Expression of IL-1β and IL-6 in ME-Treated RAW 264.7 Cells

**Figure 5:**
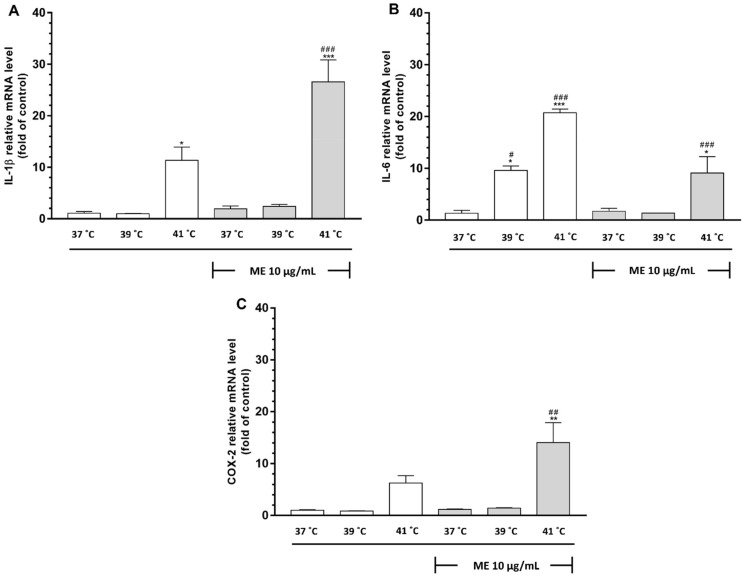
Effect of heat exposure on ME-induced mRNA expression in RAW 264.7 cells. (**A**) IL-1β, (**B**) IL-6, and (**C**) COX-2 mRNA expression was determined by quantitative real-time PCR. Data are presented as the mean ± S.E.M. of three independent experiments. Asterisks indicate statistical significance in comparison to untreated control cells and slashes indicate significance in comparison to ME-treated cells cultured at 37 °C (*** or ### *p* < 0.001, ** or ## *p* < 0.01, * or # *p* < 0.05). ME indicates mistletoe extract.

Having found a significant influence of heat on the expression of pro-inflammatory cytokines in MCF7 cells, we decided to determine whether similar effects are observed in RAW264.7 cells. We observed that cells exposed to heat at 41 °C revealed an increased expression of IL-1β in comparison to control cells (*p* < 0.05) (Figure 5A). The exposure of RAW 264.7 cells to ME did not change the mRNA expression of IL-1β in cells cultured at 37 °C in comparison to control cells (*p* > 0.05). Similarly, the exposure of ME-treated cells to heat at 39 °C did not affect this expression (*p* > 0.05). However, in cells cultured at 41 °C, we observed a significant increase in IL-1β mRNA expression (*p* < 0.001), which was about two times higher than in cells cultured at 41 °C without ME (*p* < 0.01).

The observation of not-treated with ME cells revealed that exposure to heat alone enhances IL-6 mRNA expression at both 39 °C and 41 °C compared to cells cultured at 37 °C (*p* < 0.05 and *p* < 0.001, respectively) (Figure 5B). ME did not change mRNA expression of IL-6 in RAW 264.7 cells cultured at 37 °C in comparison to control cells (*p* > 0.05). Similarly, the simultaneous treatment of RAW 264.7 cells with ME and incubation at 39 °C did not affect this expression (*p* > 0.05). However, in ME-treated cells, incubation at 41 °C triggered a significant elevation of mRNA expression in comparison to ME-treated cells cultured at 37 °C and control cells (*p* < 0.05 and *p* < 0.001, respectively).

### 2.13. ME Combined with Incubation at 41 °C Caused a Significant Increase in Expression of Cyclooxygenase-2 in RAW 264.7 Cells

Having found an enhanced expression of pyrogenic cytokines in response to heat exposure in RAW 264.7 cells, we decided to check whether COX-2, another factor involved fever mechanism, is also stimulated. In RAW 264.7 cells that were not treated with ME, we observed that exposure to heat at 39 °C did not affect COX-2 expression (Figure 5C). After heat exposure at 41 °C, RAW264.7 cells revealed an increased expression of COX-2 in comparison to control cells, but this change was statistically insignificant (*p* > 0.05). ME at 37 °C did not affect COX-2 expression in RAW 264.7 cells compared to control cells. Surprisingly, exposing the ME-treated cells to heat at 41 °C significantly enhanced this expression, whereas culturing cells at 39 °C did not change it (*p* < 0.01, and *p* > 0.999, respectively).

## 3. Discussion

Mistletoe preparations (ME) are widely used in patient-centered integrative cancer care. They induce a significant increase in beta-endorphin plasma levels; therefore, they can be used to reduce patients’ emotional stress [17]. In addition to these psychoactive properties, their immunomodulatory properties have been established [18,19]. In many patients, ME administration generates a dose-dependent fever-like reaction. Whether this reaction is important for ME therapy is unknown.

Fever is one of the most commonly recognized features of acute inflammation. There is much evidence that infectious fever acts on the immune system and may promote an anticancer response in patients [20,21,22]. However, the significance of fever following ME administration is not well understood, and what is known is based only on observational studies that do not explain the molecular consequence of the increase in body temperature. Therefore, to investigate this issue, we cultured ME-treated breast cancer cells (MCF-7, and 4T1) and immune cells (macrophages, RAW 264.7) in FRH. These conditions reflect in vitro fever-associated body temperature increases. Since it has not been established whether moderate (37.5–40.5 °C) or high (above 40.5 °C) hyperthermia is more beneficial for cancer treatment, we tested both. To the best of our knowledge, this is the first investigation examining the impact of febrile temperatures on ME-induced effects observed in normal and malignant cells.

In the current study, we investigated the cell viability, ROS generation, cell cycle distribution, and the expression of pro-inflammatory factors. We observed only slight differences between cell viability, ROS production, and cell cycle distribution in between both cancer cell lines. We found that ME in a dose of 10 μg/mL used as a monotherapy triggers only a slight decrease in the cell viability of breast cancer cells. These results are in accordance with results described by Weissenstein and colleagues [23], who found that ME only displays a dose-dependent anti-proliferative effect on breast cancer cells at concentrations ≥ 10 μg/mL. Similarly, Klingbeil and colleagues [24] found an increase in mortality of neck squamous cell carcinoma cell lines when ME was used in a dose of 300 μg/mL for 48 h. After we established that 10 μg/mL of ME is not sufficient to provoke a decrease in the viability of breast cancer cells, we utilized a surrogate of fever called fever-range hyperthermia (FRH) to assess whether heat may inhibit their proliferation. Indeed, we found that both ME-treated lines of cancer cells that were cultured at high FRH (41 °C) demonstrated a significant decrease in cell viability. In MCF-7 cells, this effect can result from excessive ROS generation, but in the 4T1 cells, this effect seems to be independent of ROS generation. ME-treated MCF-7 breast cancer cells cultured at moderate FRH (39 °C) did not display any additional ROS generation. In contrast, in ME-treated 4T1 cells, we observed a minor decrease in ROS generation after moderate FRH treatment. Additionally, we have found only a slight decrease in cell viability in both cancer cell lines. Since our control of not-treated with ME breast cancer cell lines revealed that exposure to 41 °C significantly modulates cell viability and ROS production, we suppose that fever in a range of 41 °C may trigger similar effects in cancer cells.

That ME-treated breast cancer cells react differentially to various FRH was also observed in cell cycle distribution analysis. Although 41 °C inhibits cell proliferation in comparison to 37 °C, it did not change the distribution of the cell cycle in MCF-7 cell lines, whereas at 39 °C, it induced only a slight decrease in cell proliferation that was accompanied by an increase of cells in the G1 pool. Interestingly, since we observed a significant decrease in cell viability of ME-treated 4T1 breast cancer cells cultured at 41 °C, we observed a simultaneous decrease of cells in the G1 phase, whereas in cells cultured at 39 °C, we observed only a slight decrease in cell viability and a slight decrease in the number of cells in the G1 phase. Thus, although the effect of FRH on ME-treated cancer cell viability is similar in both examined cell lines, their response in ROS generation and cell cycle distribution is slightly different.

Therapy based on ME may affect not only cancer cells but also immune cells. Therefore, in the current research, apart from breast cancer cells, we also investigated macrophages treated with FRH and ME. Our investigation revealed that macrophages responded to ME and FRH differently than breast cancer cells. The incubation of macrophages with ME resulted in the cell viability decrease, although the production of ROS and distribution of cells throughout the cell cycle did not change in comparison to control cells. This decrease was prevented by additional treatment of cells with FRH at 39 °C as well as 41 °C. Although, both types of hyperthermia induced an increase in proliferation of ME-treated cells in comparison to ME-treated cells cultured at 37 °C, the distribution of cell cycle differed significantly and was temperature dependent. At 39 °C, a significant increase of G1 was observed, whereas at 41 °C, we observed an increase of cells in the G2/M pool. This observation clearly shows that FRH creates conditions that favor the proliferation of macrophages, although the cell cycle distribution of cells cultured at these two thermal conditions differed significantly. Moreover, we observed that both ME-treated and not-treated with ME macrophages that were exposed to 41 °C displayed a significant increase in ROS generation along with increased proliferation. It is known that macrophages produce intracellular ROS that are involved in the phagocytic process. There is also a notion that macrophage-generated ROS are essential for the uptake and clearance of apoptotic cells [25,26]. Furthermore, ROS can function as second messengers and modulate cell functions by activation of cell signaling pathways in response to stimulation with various agents [25].

In the next step of our research, we investigated whether FRH modifies mRNA expression of pro-inflammatory cytokines in both ME-treated cell types. Although we found that 10 µg/mL of ME triggered only a slight decrease in the cell viability of breast cancer cells, this dose of ME significantly affects IL-1β and IL-6 mRNA expression. Interestingly, the expression of cytokines in both types of breast cancer cell lines differed significantly e.g., ME significantly enhanced the expression of IL-1β and IL-6 genes in MCF-7 cells, whereas we did not observe such an effect in 4T1 cells. Furthermore, FRH at 41 °C decreases the IL-6 mRNA level in ME-treated MCF-7 cells, whereas in the ME-treated 4T1 cell line, a significantly increase in IL-6 mRNA level was observed. Additionally, we have found that a combination of ME and mild FRH (39 °C) reduced this expression, and the combination of ME and high FRH (41 °C) inhibited the expression of these cytokines completely in MCF-7 cells. Thus, FRH inhibits IL-1β and IL-6 mRNA expression in these breast cancer cells in a temperature-dependent manner. In ME-treated 4T1 cells, we did not observe an increase in the expression of IL-1β and IL-6 mRNA; even additional treatment with moderate FRH (39 °C) did not change it. Nevertheless, high FRH (41 °C) decreases the IL-1β mRNA level, and it surprisingly increases the level of IL-6 mRNA in ME-treated 4T1 breast cancer cells. Furthermore, our control group, not-treated with ME, revealed an increase in IL-6 mRNA expression that was temperature-dependent. We suppose that these differences in cytokines expression can be associated with different aggressiveness and characteristic features of both examined breast cancer cell lines. 4T1 cells are considered to be highly aggressive, triple negative cells [27]. In contrast, MCF-7 cells are poorly invasive [28], with estrogen and progesterone receptors [29]. This issue needs further research, since the production of pro-inflammatory cytokines by cancer cells is considered harmful [30]. Our research revealed also that not-treated with ME breast cancer cells display an increased mRNA expression of IL-1β and IL-6 in response to heat at 39 °C alone but not to 41 °C.

Unlike cancer cells, the co-treatment of macrophages with ME and high FRH (41 °C) induced a strong up-regulation of IL-1β and IL-6 mRNA expression. These findings together with the observation of increased ROS production in ME-treated macrophages cultured at 41 °C suggests that there is a switch toward the M1 phenotype [26,31]. It is believed that this type of macrophage is implicated in the effective elimination of tumor cells [32]. Thus, the finding that FRH may orchestrate the switch toward M1 is of great significance. Najafi and colleagues [33] postulated that macrophage switching toward an anti-inflammatory M1 phenotype could be used as an adjuvant with other approaches, including radiotherapy and immune checkpoint blockades, such as anti-PD-L1/PD-1 strategies. Our findings show that this phenotype can be induced by the exposure of ME-treated macrophages to FRH at 41 °C.

Having found an enhanced expression of pro-inflammatory cytokines in ME-treated macrophages, we additionally measured the mRNA expression of COX-2, which is not only another factor involved in inflammation but is also an M1 macrophage marker [34]. We observed that only high FRH (41 °C) increased COX-2 mRNA expression. Since our research revealed that incubation at 41 °C induces an increase in the expression of pyrogenic cytokines and COX-2 in macrophages, we speculate that this FRH triggers signaling pathways that are similar to those induced by pathogens. Additionally, this finding suggests that heat in a range of 41 °C acts as an adjuvant of inflammatory response. These hypotheses are supported by observations demonstrating the role of heat shock proteins in inflammation. It is commonly known that intracellular HSP70 acts as a chaperone that exerts anti-apoptotic and cytoprotective actions [35], and other research has shown that extracellular HSP70 is also an immunomodulator, which, similarly to lipopolysaccharide, acts as a Toll-like receptor agonist [36,37]. Therefore, further research is needed to determine whether the increase in the expression of pro-inflammatory cytokines and COX-2 observed in our investigation is indeed a consequence of heat shock proteins released after FRH treatment.

Given that the expression of genes such as IL-1β, IL-6, and COX-2 is nuclear factor-κB (NF-κB) dependent, we hypothesize that in poorly invasive cancer cells, the heat is an inhibitor of NF-κB, which acts in a dose-dependent manner. Since we observed different effects in highly invasive breast cancer cells, the role of heat remains unclear. Nevertheless, in macrophages, the heat at 41 °C activates the NF-κB-dependent signaling pathway, leading to the production of IL-1β, IL-6, and COX-2. Our opinion is consistent with conclusions by Harper and colleagues [38], who showed that temperature regulates the inflammatory response through the modulation of NF-κB signaling and its downstream mediators.

## 4. Materials and Methods

### 4.1. Mistletoe Extract (ME)

Commercially available, standardized ME (brand name Iscador Qu; Weleda AG, Schwäbisch Gmünd, Germany; PZN 1386131) from mistletoe grown on oak trees was used at a dose of 10 μg/mL, which corresponds to doses used for intravenous applications [39].

### 4.2. MCF-7 Cell Line

The human breast cancer cell line MCF-7 was obtained from the European Collection of Cell Cultures (Lot. 13K023; Salisbury, UK). Cells were cultured in RPMI 1640 culture medium supplemented with 1% antibiotic mixture (100 IU/mL penicillin and 100 µg/mL streptomycin), 10% heat-inactivated fetal bovine serum, and 1× non-essential amino acids (all reagents were from Sigma Aldrich, Darmstadt, Germany). Cells were grown under stable thermal conditions (37 °C), in a humidified atmosphere of 5% CO_2_. The culture medium was changed every second day, and cells were removed from culture flasks or plates using 0.25% trypsin–EDTA solution (Sigma Aldrich).

#### 4T1 Cell Line

The murine breast cancer 4T1 cell line was purchased from the American Type Culture Collection (Manassas, VA, USA). Cells were cultured in Dulbecco’s modified Eagle’s medium supplemented with 10% fetal bovine serum (FBS), 100 µg/mL streptomycin, and 100 IU/mL penicillin (all reagents were from Sigma-Aldrich) at 37 °C in a humidified atmosphere with 5% CO_2_. The culture medium was changed every 2–3 days. Cells were passaged and/or collected using 0.25% trypsin–EDTA solution (Sigma-Aldrich) when reaching 70–80% of confluency.

### 4.3. RAW 264.7 Cell Line

The murine macrophage cell line RAW 264.7 was obtained from the European Collection of Cell Cultures (cat. No. 91062702). RAW 264.7 cells were cultured in DMEM culture medium (Sigma Aldrich) supplemented with 10% heat-inactivated FBS and a mixture of antibiotics (100 µg/mL streptomycin and 100 IU/mL penicillin). Cells were maintained under controlled conditions at 37 °C and in a humidified atmosphere with 5% CO_2_. The culture medium was changed every second day. To collect the cells, cells were rinsed, and a cell scraper was used to remove adherent cells from the culture plates or culture flasks.

### 4.4. Heat Exposure and Treatment with Mistletoe Extract

After collection of the cells, the total number of viable cells was determined by trypan blue exclusion, using the LUNA^TM^ automated cell counter (Logos Biosystems, Annandale VA, USA). The cells were seeded in 96-well, 12-well, or 6-well plates at a density of 5 × 10^3^/well, 2 × 10^5^/well, or 3 × 10^5^/well, respectively, depending on the experiments. Following an overnight preincubation at 37 °C, the cells were co-treated with ME (Iscador Qu, Weleda) at a concentration of 10 µg/mL and cultured at 37 °C, 39 °C, or 41 °C for 24 h. Cells cultured at 37 °C and not treated with ME were included as the control cells.

### 4.5. Assessment of Cell Viability Using the MTT Assay

The viability of the cells (MCF-7,4T1 and RAW 264.7) treated with ME and exposed to various heat conditions was determined using the MTT viability assay. In living cells, the absorbed yellow 3-(4,5-dimethyl thiazolyl)-2,5-diphenyl-tetrazolium bromide (MTT; Sigma Aldrich) is reduced by mitochondrial dehydrogenase to a blue formazan product. Briefly, the cell supernatants were removed, the MTT solution was added (100 μL/well; final concentration of 0.5 mg/mL), and plates were incubated for 3 h at 37 °C, 5% CO_2_ in a humidified atmosphere. After the incubation period, the supernatants were removed, and 100 µL of dimethyl sulfoxide was added to dissolve the formazan crystals. The plates were mixed horizontally for 5 min, and the absorbance was measured at 570 nm (with a reference wavelength of 630 nm) using a Synergy HT Multi-Mode Microplate Reader (BioTek Instruments, Winooski, VT, USA). Cell viability was calculated as the percentage of the absorbance 570/630 nm ratio in experimental wells compared to the control wells.

### 4.6. Reactive Oxygen Species Determination Using Flow Cytometry

Cellular reactive oxygen species (ROS) level was measured by a technique that converts carboxy-H_2_DCFDA (5(6)-carboxy-2′,7′-dichlorofluorescein diacetate, Sigma Aldrich) into a green fluorescence dye 2′,7′–dichlorofluorescein (DCF). Briefly, cultured cells were counted, washed with phosphate-buffered saline (PBS), and resuspended in carboxy-H2DCFDA at a final concentration of 10 μM in regular culture medium with reduced serum (2%). Cells were incubated in the dark for 30 min in a conventional incubator at 37 °C. After the incubation period, cells were washed with PBS, seeded in six-well plates at the density of 3 × 10^5^ cells/well, and treated with ME at a final concentration of 10 µg/mL in regular culture medium with reduced serum (2%). These cells were cultured for 24 h at different thermal conditions. Next, the cells were collected as described above, washed twice with PBS, and resuspended in PBS. ROS level was assessed by immediately analyzing cells using flow cytometry (FL1 channel/green fluorescence) BriCyte E6 flow cytometer (Mindray, Shenzhen, China). Results are presented as a percent change in comparison to control cells (cultured at 37 °C and not treated with ME).

### 4.7. Cell Cycle Analysis by Flow Cytometry

The cell cycle was determined by the quantitation of DNA content using the nucleic acid stain propidium iodide followed by flow cytometry analysis. Propidium iodide (PI) is a fluorescence dye that binds both types of nucleic acids, DNA and RNA, proportionally to the amount of material present in the nucleus. Cells were seeded at a density of 3 × 105 in six-well plates, as described above. Following overnight preincubation, cells were treated with ME and exposed to various temperature conditions for 24 h. After treatment, the culture media was removed, and cells were collected as described above. Cell cycle analysis was carried out using a CellCycleFlowEx^®^ Kit (Exbio, Vestec, Czech Republic) according to the manufacturer’s instructions. Cells were stained for 30 min with PI, and RNA was digested using RNAse. Next, flow cytometry analysis was conducted within 4 h using a BriCyte E6 flow cytometer (Mindray, Shenzhen, China). The cell cycle distribution was presented as the percentage of cells containing 2n (G1 phase), 4n (G2 and M phase), and between 2n and 4n (S phase), as determined by PI staining.

### 4.8. Quantification of Cytokines and COX-2 mRNA Expression

Following heat exposure and ME treatment, determination of IL-1β, IL-6, and COX-2 mRNA expression was conducted using two-step RT-qPCR. The extraction of total mRNA was conducted according to the Chomczynsky and Sacchi method [40]. PureZOL™ RNA Isolation Reagent (Bio-Rad, Hercules, CA, USA) was used according to the manufacturer’s instructions. The final concentration of total mRNA in samples was measured using a Take3 Micro-Volume Plate for the Synergy HT Multi-Mode Microplate Reader. Synthesis of cDNA was carried out on 300 ng of total RNA using iScript™ Reverse Transcription Supermix for RT-qPCR (Bio-Rad), according to the manufacturer’s instructions. Quantitative real-time PCR was performed according to the manufacturer’s protocol in a final volume of 10 µL containing cDNA, iTaq Universal SYBR^®^ Green Supermix (Bio-Rad) and PrimePCR™ SYBR^®^ Green Assay to amplify IL-1β (Unique Assay ID: qMmuCID0005641 and qHsaCID0022272), IL-6 (Unique Assay ID: qMmuCID0005613 and qHsaCED0044677), COX-2 (Unique Assay ID: qHsaCED0042341), and GAPDH (Unique Assay ID: qMmuCED0027497 and qHsaCED0038674) as a reference gene using the CFX Connect Real-Time PCR Detection System (Bio-Rad). All assays were obtained from Bio-Rad. Test samples were run in triplicates. Each reaction was repeated at least two times. Melt curve analysis was performed on a Bio-Rad CFX96 as a control for specificity of the products. Standard curves were prepared for target (IL-1β, IL-6, and COX-2) and reference (GAPDH) genes. Calibrator-normalized relative quantification was carried out using CFX Manager Software 3.1 (Bio-Rad).

### 4.9. Statistical Analysis

All values are reported as mean ± standard error of the mean (SEM) of three independent experiments. Statistical significance was determined using analysis of variance (two-way ANOVA) followed by the Tukey test at a critical value of *p* < 0.05.

## 5. Conclusions

In conclusion, FRH significantly modulates ME-induced effects in both cancer cell lines, as well as in immune cells; therefore, we think that the increase in body temperature observed in some patients after treatment with immunomodulators may be of great significance not only for ME therapy, but for immunotherapies in general. Thus, if fever is not activated after ME administration, whole body FRH can be used as an alternative to fever [41]. However, this procedure needs careful consideration of all consequences, including those pointed in this article. We suggest that more attention should be directed to the role of temperature in the regulation of immune and cancer cells, especially when they are treated with immunomodulators.

## Data Availability

Data available in a publicly accessible repository (https://repod.icm.edu.pl/, accessed on 8 June 2021).
